# Modulation of ankle antagonist co-activation during the transition from upright standing to gait and to sit in post-stroke subjects

**DOI:** 10.1080/07853890.2021.1896620

**Published:** 2021-09-28

**Authors:** Edgar Ribeiro, Augusta Silva, Liliana Pinho, Rubim Santos, Francisco Pinho, Andreia S. P. Sousa

**Affiliations:** a Center for Rehabilitation Research, School of Health, Polytechnic of Porto, Porto, Portugal; b CESPU, Institute of Research and Advanced Training in Health Sciences and Technologies, V. N. Famalicão, Portugal; c Polytechnic Institute of Cávado and Ave, Barcelos, Portugal

## Abstract

**Introduction:**

Antagonist co-activation represents a neuronal command for the modulation of muscle synergies with postural control purposes [1], probably assuming a key role in the characterisation of tonus dysfunction in post-stroke subjects. This study aims to evaluate the ankle antagonist co-activation during different functional tasks in post-stroke subjects.

**Materials and methods:**

A cross-sectional study was performed in eight participants (age = 43.00 ± 10.63 years; median ± interquartile range) who had a subcortical ischaemic stroke in the middle cerebral artery territory for at least 6 months. The study was approved by the local ethics committee and implemented in a research centre. Antagonist co-activation between tibialis anterior (TA) and soleus (SOL) and between TA and gastrocnemius medialis (GM) of the ipsilesional (IPSI) and contralesional (CONTRA) limbs was calculated through electromyographic signals collected during upright standing and postural phases of gait initiation and stand-to-sit, according to the methods proposed by Ribeiro [2].

**Results:**

The CONTRA limb presented decreased values in TA/SOL pair during upright standing and increased values in both muscle pairs during gait initiation compared to the IPSI limb (Table 1). No significant differences were found between tasks (Table 1).

**Discussion and conclusions:**

The IPSI and CONTRA limbs presented increased antagonist co-activation when an adequate antigravity function and the coordination of the tibia forward rotation are required, respectively. The comparison of these values with that obtained by healthy subjects [3,4] seems to point to a bilateral postural control dysfunction in post-stroke subjects related to tonus modulation deficits that should be addressed in neurorehabilitation. Future studies with a higher sample are required to extend the results.

**Table 1.** Median (Md) and interquartile range (IQR) of antagonist co-activation during functional tasks and p-values obtained from inter-limb and inter-task comparisons.

Muscle pair	Limb	Functional task						*p*-value
		Upright standing		Gait initiation		Stand-to-sit		
		Md (IQR)	*p*-value	Md (IQR)	*p*-value	Md (IQR)	*p*-value	
TA/SOL	IPSI	96.8 (38.8)	.039	71.1 (10.4)	.016	80.5 (15.5)	.250	.964
	CONTRA	50.9 (24.7)		100.9 (5.2)		94.1 (7.6)		.112
TA/GM	IPSI	88.9 (30.9)	.742	70.0 (7.6)	.016	77.0 (35.4)	.148	.964
	CONTRA	78.1 (20.9)		104.9 (1.7)		96.3 (4.9)		.305

## References

[1] Latash ML, Huang X. Neural control of movement stability: lessons from studies of neurological patients. Neuroscience. 2015;301:39–48. 26047732 10.1016/j.neuroscience.2015.05.075 PMC4504804

[2] Ribeiro E, Silva A, Pinho L, et al. Correlation between ankle stiffness and antagonist co-activation in post-stroke subjects. IJHFE. 2019;6(4):331–354.

[3] Sousa ASP, Macedo R, Santos R, et al. Influence of prolonged wearing of unstable shoes on upright standing postural control. Hum Mov Sci. 2016;45:142–153. Feb 26638047 10.1016/j.humov.2015.11.015

[4] Silva A, Sousa ASP, Silva C, et al. Ankle antagonist coactivation in the double-support phase of walking: stroke vs. healthy subjects. Somatosensory Motor Res. 2015;32(3):153–157. 10.3109/08990220.2015.1012492 26290312

